# A Case of Microscopic Thymoma and Myasthenia Gravis: Key Points for Clinical Features and Surgical Management

**DOI:** 10.1155/cris/1354084

**Published:** 2026-06-28

**Authors:** Nicola Rotolo, Francesca Franzi, Elena Asteggiano, Alberto Colombo, Stefano La Rosa, Elisa Nardecchia

**Affiliations:** ^1^ Thoracic Surgery Unit, Department of Medicine and Technological Innovation–Research Center of Minimally Invasive Surgery, Università degli Studi dell’Insubria, Varese, Italy, uninsubria.it; ^2^ Unit of Pathology, Department of Oncology, Azienda Socio Sanitaria Territoriale, Varese, Italy; ^3^ Thoracic Surgery Unit, Department of Medicine and Surgery–Research Center of Thoracic Surgery, Università degli Studi dell’Insubria, Varese, Italy, uninsubria.it; ^4^ Unit of Pathology, Department of Medicine and Technological Innovation, Hereditary Cancer Research Center, Università degli Studi dell’Insubria, Varese, Italy, uninsubria.it

**Keywords:** case report, microscopic thymoma, myasthenia gravis, thymic nodular hyperplasia, thymothymectomy

## Abstract

Microscopic thymoma (MT) is defined as solitary or multifocal small thymic epithelial nests <1 mm in diameter, occurring in approximately 15% of patients with myasthenia gravis (MG) and sometimes associated with thymic cysts. Its preoperative diagnosis is challenging due to its small size, and computed tomography (CT) scans are unable to detect it. MTs are considered incipient or precursor lesions of thymoma, detectable only via microscopic examination. We report a case of MT identified pathologically in the thymic tissue surrounding a thymic cyst in a 52‐year‐old female hospitalized for drooping eyelids and muscle weakness. A mediastinal CT scan revealed an enlarged thymic gland with an associated cyst in the anterior mediastinum. Due to symptomatic disease, we performed a right video‐assisted thoracoscopic thymectomy. No macroscopic tumor was observed, but histopathological analysis revealed an acquired multilocular thymic cyst with multiple thymic epithelial islands (MTs) in the surrounding thymic tissue. All previous cases reported, except one, were symptomatic for MG and none were diagnosed before surgery. The morphological pattern of MT differs from conventional thymoma, and the latest WHO classification categorizes it as thymic nodular hyperplasia. To maximize the detection of MT nests, complete resection and thorough histopathological examination of the thymic gland and surrounding tissue are essential, even in the absence of macroscopic lesions. Here, we propose key points for the evaluation and management of MT.

## 1. Introduction

Microscopic thymoma (MT) is defined as a thymic epithelial proliferation smaller than 1 mm, typically detected incidentally in patients undergoing thymectomy for myasthenia gravis (MG). Due to its small size, it is rarely detected preoperatively, and its clinical significance remains a topic for discussion. MT is considered a precursor lesion rather than a fully developed thymoma, although its exact pathogenesis remains unclear, often associated with MG and sometimes with thymic cysts. In this report, we describe a case of MT associated with a thymic cyst in a patient with MG, providing a literature review to contextualize the findings and discuss clinical implications. The aim of this paper is to define certain key points that allow for the identification of MT outcomes.

## 2. Case Presentation

A 52‐year‐old female was admitted for progressive ptosis and muscle weakness, which was consistent with ocular MG at presentation. Neurological evaluation raised the suspicion of MG, prompting further investigations. At diagnostic workup, a computed tomography (CT) chest scan revealed an enlarged 70 mm × 40 mm thymic gland with a cystic component in the anterior mediastinum (Figure 1). A positron emission tomography (PET) showed a low FDG uptake of the lesion. Although both anti‐acetylcholine receptor (AChR) and muscle‐specific receptor tyrosine kinase (anti‐MuSK) antibodies were within normal limits, the clinical presentation was highly suggestive of MG. A neurologist established the diagnosis of seronegative MG based on clinical criteria. No electrophysiological studies (repetitive nerve stimulation or single‐fiber electromyography) or pharmacological tests (edrophonium) were performed. Nevertheless, the patient was referred to a Multidisciplinary Tumor Board (MDT), which identified the presence of a significant thymic lesion in a symptomatic patient as the surgical indication. The decision was made to proceed directly to video‐assisted thoracoscopic thymectomy for both diagnostic and therapeutic purposes. No preoperative immunosuppressive therapy (corticosteroids) or plasmapheresis was administered.

**Figure 1:**
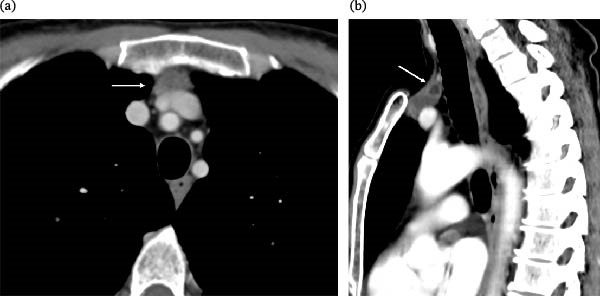
Axial and sagittal chest computed tomography showed (a) a small not‐involuted thymic gland (arrow) with (b) associated pretracheal cystic lesion (arrow).

After selective bronchial intubation with a double lumen, we performed a right triportal video‐assisted thoracoscopic surgery (VATS) using one 10 mm optical port and two 5 mm instrument ports. CO_2_ insufflations were also used. An extended thymectomy was performed, including complete resection of the thymic gland and the anterior mediastinal and perithymic fatty tissues between both phrenic nerves, from the cardiophrenic angles up to the lower cervical region. The right‐sided approach was selected because the lesion was small, noninvasive on imaging, and located predominantly on the right side of the anterior mediastinum. CO_2_ insufflation allowed adequate exposure of the contralateral mediastinal pleura and cervical thymic horns.

No macroscopic tumor was observed intraoperatively. One 12 French chest drainage was taken in the thorax. The postoperative course was uneventful, and the patient was discharged home on the fourth postoperative day with resolution of myasthenic symptoms. Figure 2 shows a thymic cyst and a small solid nodule between the upper horns. No other macroscopic lesions were identified.

**Figure 2:**
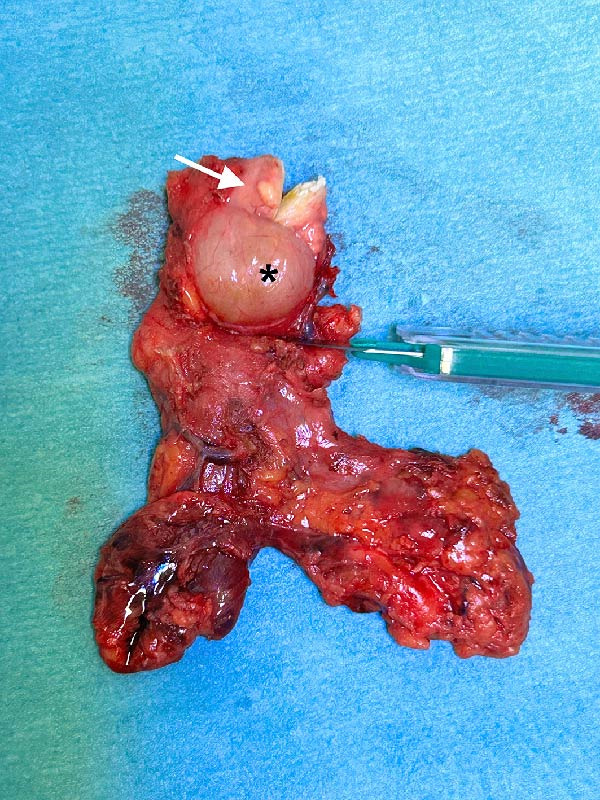
Gross image of resected thymic specimen. The multilocular cyst (black asterisk) and a small solid nodule (white arrow) are visible between the upper horns. No other macroscopic lesions were identified.

The examination of the specimen revealed an acquired multilocular thymic cyst and multiple small islands (<1 mm in diameter) of spindle thymic epithelial cells with no atypia within the surrounding thymic tissue, consistent with MT (Figure 3).

**Figure 3:**
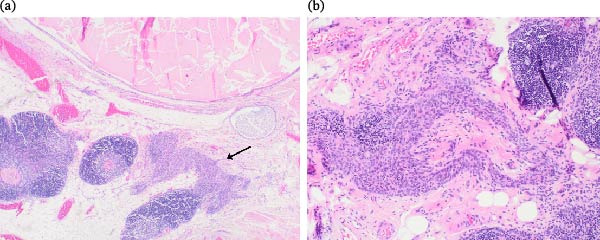
(a) Thymic cyst and small thymic epithelial nodules (arrow) in the surrounding thymic tissue (HE 20x) and (b) thymic epithelial islands (HE 100x).

At 29 months of follow‐up, the patient remains in good clinical condition.

## 3. Discussion

MT remains a rare entity, with limited cases reported in the literature. First described by Rosai et al. in 1976 [1], only 25 cases have been reported to date, including our report (Table 1), occurring in a range of 3%–15% of resected thymus and surrounding tissue, and its diagnosis is only at microscopic specimen evaluation. Although the term “microscopic thymoma” is retained in the title of this report for consistency with the existing clinical literature, the current WHO classification (fifth edition, 2021) [13] categorizes this lesion under the broader entity of thymic nodular hyperplasia [6] because there are not sufficient data regarding its role in the genesis of thymoma.

**Table 1:** Medical and pathological records of 25 cases of microscopic thymoma published in the literature.

Authors	Cases	Sex	Age	Myasthenia	AChR	MuSK	Surgical approach
Poulard et al. [2]	1	f	59	NA	NA	NA	NA
Pescarmona et al. [3]	3	f	32	NA	NA	NA	NA
		m	38				
		f	42				
Puglisi et al. [4]	1	m	56	+	+	NA	VATS
Chalabreysse et al. [5]	3	f	58	+	NA	NA	Sternotomy
		m	29	+			Sternotomy
		f	38	+			Manubriotomy
Cornea et al. [6]	1	f	30	+	NA	NA	VATS
Vaideeswar et al. [7]	4	f	17	+	+	NA	NA
		m	23	+	+		
		f	38	+	+		
		m	46	+	+		
Yu et al. [8]	3	NA	NA	NA	NA	NA	NA
Fukuhara et al. [9]	5	m	31–64	+	+	NA	Sternotomy
		m		+	+		Sternotomy
		m		+	+		Sternotomy
		f		+	+		Sternotomy
		f		+	+		Sternotomy
Furuya et al. [10]	1	m	75	−	NA	NA	VATS + sternotomy
Banerjee et al. [11]	1	f	64	+	−	+	Sternal split
Komatsu et al. [12]	1	f	81	−	NA	NA	RATS
Rotolo, 2026 [^a^]	1	f	52	+	−	−	VATS

*Note*: “+” indicates positive and “−” indicates negative.

Abbreviations: AChR, autoantibodies against the muscle acetylcholine receptor; MuSK, antibodies against the muscle specific receptor tyrosine kinase. NA, not available; RATS, robotic‐assisted thoracoscopic; VATS, video‐assisted thoracoscopy.

^a^Present study.

The MT has a different morphology than that of conventional thymoma, with a lack of lobulation, perivascular spaces, medullary differentiation, and capsule. In our case, the microscopic epithelial nests were predominantly composed of spindle‐shaped epithelial cells, similar to previously reported descriptions of MT [14]. It is defined as a very rare other thymoma entity such as microthymoma, metaplastic thymoma, sclerosing thymoma, and lipo‐fibroadenoma [1] for which there are no recorded fatal outcomes [13]. Fukuhara et al. [9] analyzed 18 cases, emphasizing their frequent association with MG; furthermore, Komatsu et al. [12] described a case without MG. The case described by Komatsu et al. [12] was operated on due to an enlarging thymic cyst causing mild chest discomfort without evidence of MG. This represents the only reported case of MT resected for a nonmyasthenic indication [12].

We acknowledge the absence of electrophysiological confirmation of MG as a limitation of this case report. In routine clinical practice, current guidelines recommend neurophysiological testing (RNS or SFEMG) in seronegative patients. However, in this specific case, the presence of a radiologically evident thymic lesion provided an independent indication for surgery, and the multidisciplinary team elected to proceed without further diagnostic testing. This approach was justified by the dual goal of thymectomy: pathological diagnosis of the mediastinal lesion and potential improvement of myasthenic symptoms.

Notably, in seronegative MG, thymus‐driven immune dysregulation (including T‐cell–mediated mechanisms or low‐affinity antibodies not routinely detected) has been proposed as a pathogenic mechanism, particularly in the presence of thymic abnormalities. This supports the biological rationale for thymectomy even in antibody‐negative patients with thymic lesions.

Nevertheless, we emphasize that electrophysiological confirmation remains the standard of care in seronegative MG when surgical indication is not already established by imaging findings. Precisely because of its multifocal nature, in the absence of macroscopically detectable nodular lesions, it is useful to discuss the case with pathologists to search for possible microscopic foci of thymoma in the surgical specimen and the surrounding resected tissues, especially in MG patients. Its peculiar characteristic, which could lead to a misdiagnosis, is that it is not detected on imaging preoperatively (both CT scan and PET scan). Moreover, even after thymus removal, the gland shows no evidence of nodules or tumors, and the diagnosis is based entirely on microscopic findings. In our case, the CT thorax scan showed a thymic cyst within a noninvoluted thymic gland. Acquired thymic cysts have been described in association with thymic epithelial proliferations and may reflect an underlying inflammatory or immune‐mediated process.

According to NCCN guidelines, MRI is recommended for the differential diagnosis of thymic lesions, particularly to distinguish cystic thymoma from benign thymic cysts. In our institution, MRI was not performed because the lesion lacked solid components on CT, and a benign cyst was presumed. In retrospect, MRI might have provided additional characterization, although MTs remain below the resolution of any current imaging modality.

A full thymectomy is the standard approach for symptomatic patients, despite imaging not revealing any thymic lesions preoperatively, associated with complete removal of perithymic fat tissue. Some thoracic surgeons prefer the noninvasive approaches (VATS or robotic‐assisted thoracoscopy [RATS]) to resect the thymus, whereas others still consider median sternotomy the reference approach for MG patients to achieve complete resection of thymic and perithymic tissue. The choice between a minimally invasive approach and sternotomy depends on several factors, including the size and invasiveness of the lesion and the need for an extended thymectomy. Although median sternotomy has historically been considered the reference approach for thymectomy in MG patients, several minimally invasive series have demonstrated that a complete thymectomy can be safely achieved through unilateral VATS in selected non‐invasive thymic diseases when performed in experienced centers. The minimally invasive thoracic procedures are those performed through intercostal, subxiphoid, transcervical, and subcostal incisions [15, 16]. In our case, due to the small size of the thymic gland, the absence of radiological signs of invasion, and the presence of a localized cystic lesion, we opted for a right‐sided VATS‐extended thymectomy, even though other colleagues have mainly used a sternotomy to treat similar cases (Table 1).

A significant clinical implication of this case is that current management guidelines do not routinely recommend thymectomy for seronegative or ocular MG. Consequently, MTs (which are invisible to imaging and only detectable on histopathological examination) may be significantly underdiagnosed in this patient population. This represents a potential diagnostic pitfall in clinical practice. Our case suggests that, in selected patients, (pharmacoresistant seronegative MG), a lower threshold for considering thymectomy might be justified as symptomatic improvement can be achieved despite negative antibodies and normal preoperative imaging. Further studies are needed to determine the true prevalence of MT in seronegative MG patients and to clarify whether these lesions have the same clinical significance as those in seropositive patients, although this remains a rare entity.

From the analysis of the few cases reported in the literature, we can outline several key points that summarize the outcomes of MT, which may help guide the diagnostic and therapeutic pathway: (1) preoperative diagnosis: imaging modalities, including CT scan and PET, often do not detect MT due to their small size. (2) Histological classification: WHO classifies MT as nodular hyperplasia of the thymic epithelium, differing from conventional thymomas. (3) Surgical management: complete thymectomy remains the standard approach for both therapeutic and diagnostic purposes. (4) Clinical implications: while often asymptomatic, MT may be precursors to larger thymomas, warranting long‐term surveillance, although this has not yet been proven.

Further studies are needed to determine the long‐term clinical outcomes and optimal management strategies for MTs.

## Author Contributions

Nicola Rotolo was involved in the conception of the work, final approval, literature search, and drafting of manuscript. Francesca Franzi, Elena Asteggiano, and Alberto Colombo did the collection and analysis of data for the manuscript. Stefano La Rosa and Elisa Nardecchia were involved in the critical review of the manuscript.

## Funding

No funding was received for this manuscript. Open access publishing facilitated by Universita degli Studi dell′Insubria, as part of the Wiley ‐ CRUI‐CARE agreement.

## Ethics Statement

Written informed consent was obtained from the patient for the publication of this anonymized case report and any accompanying images.

## Conflicts of Interest

The authors declare no conflicts of interest.

## Data Availability

Clinical data of the case are stored in the Department of Computer.
